# Comparative quantitation of aquaporin-2 and arginine vasopressin receptor-2 localizations among chronic kidney disease and healthy kidney in dogs

**DOI:** 10.14202/vetworld.2021.2773-2781

**Published:** 2021-10-27

**Authors:** Pitchaya Matchimakul, Wanpitak Pongkan, Piyamat Kongtung, Raktham Mektrirat

**Affiliations:** 1 Department of Veterinary Bioscience and Veterinary Public Health, Faculty of Veterinary Medicine, Chiang Mai University, Chiang Mai 50100, Thailand; 2 Integrative Research Center for Veterinary Circulatory Sciences, Faculty of Veterinary Medicine, Chiang Mai University, Chiang Mai 50100, Thailand; 3 Central Laboratory, Faculty of Veterinary Medicine, Chiang Mai University, Chiang Mai 50100, Thailand.

**Keywords:** aquaporin 2, canine, chronic kidney disease, immunohistochemistry, vasopressin receptor 2

## Abstract

**Background and Aim:**

Aquaporin-2 (AQP2) and arginine vasopressin receptor-2 (AVPR2) are proteins that control water homeostasis in principal cells. Chronic kidney disease (CKD) is defined as the impairment and irreversible loss of kidney function and/or structure, which causes water imbalances and polyuria. The study aimed to know the expression of AQPs and AVPR2 in the kidneys of a canine with CKD.

**Materials and Methods:**

The kidneys were collected from two dog carcasses from Small Animal Teaching Hospital, Faculty of Veterinary Medicine, Chiang Mai University. The kidney tissue was prepared for immunohistochemistry and investigated the expression and localization of tissue’s AQP2 and AVPR2. For statistical analysis, the Mann–Whitney U-test was applied to the data.

**Results:**

By immunohistochemistry, AQP2 was expressed strongly in the basolateral and apical membranes of the principal cells, whereas AVPR2 was localized in the principal cell’s basolateral membrane in both renal cortex and renal medulla. In the normal kidney, the semi-quantitative immunohistochemistry for the percentage of protein expression of AQP2 and AVPR2 was 5.062±0.4587 and 4.306±0.7695, respectively. In contrast, protein expression of AQP2 and AVPR2 in CKD was found to be 1.218±0.1719 and 0.8536±0.1396, respectively. The data shows that the percentage of AQP2 and AVPR2 expression was decreased, corresponding to a 4-fold and 5-fold in CKD (p<0.001).

**Conclusion:**

Our findings revealed that CKD was a marked decrease in AQP2 and AVPR2 expression. The central role of specific AQP2 and AVPR2 in regulating water homeostasis will provide correlations in case of CKD with polyuria.

## Introduction

Chronic kidney disease (CKD) is a typical disease in senior dogs and cats. The estimated CKD incidence in dogs and cats is 0.5-1.5%, with more than 10% of dogs and 30% of cats over the age of 15 being diagnosed with CKD [1,2]. CKD is defined as impairment and irreversible renal dysfunction and/or structure that results in progressive disease [3]. In the macroscopic, the kidney with CKD often palpates thin irregularly, and kidney size is small, as confirmed by abdominal radiography and ultrasound; conversely, renal neoplasia, pyelonephritis, or ureteral obstruction can occasionally result in large kidney size [2,3]. The microscopic appearance of the “end-stage kidney” in CKD can be broadly classified as glomerular or tubulointerstitial disease [4]. The cortex is fibrotic, the glomeruli are sclerosis, there are chronic inflammatory cell infiltrates, interstitial fibrosis, and the arteries are thickened [4-6]. Renal tubules are dilated and filled with pink casts, give the appearance of “thyroidization” (colloid-like hyaline cast formation) [7]. Azotemia, uremia, proteinuria, hypertension, polyuria, and water and electrolyte imbalance were the clinical signs [1]. The most common disorder caused by the kidney’s failure to conserve water is polyuria [8]. These could be related to a protein that functions as a water channel.

Aquaporins (AQPs) are transmembrane proteins, which assume a fundamental part in the penetrable choice of water and other little atoms, such as glycerol and urea [9]. In addition, it too plays a key part in water homeostasis in reaction to expanded plasma arginine vasopressin levels [10,11]. Previous research has shown that AQP2 is primarily found in the apical plasma membrane and intracellular vesicles [12,13]. To enhance the activity of adenylate cyclase, arginine vasopressin binds to the arginine vasopressin receptor-2 (AVPR2), which is found on the basolateral membrane of the principal cells in the renal collecting duct [14,15]. The interaction between arginine vasopressin and AVPR2 may result in an increase in protein kinase A (PKA) activation, intracellular Cyclic adenosine monophosphate (cAMP) generation, phosphorylation of AQP2, and AQPs trafficking through the apical plasma membrane, resulting in increased water permeability of the principal cells [16,17]. Mutations or dysfunction of AQP2 and AVPR2 could cause nephrogenic diabetes insipidus (NDI), which disrupted water balance and resulted in the excretion of large amounts of dilute urine [18,19]. Previous research has shown that AQP2 inadequacy can affect water balance in the kidney, which is common in humans and animals such as dogs, rats, mice, horses, and young beef cattle [1,20-23]. Furthermore, the structure of the canine kidney is unipyramidal, which differs from that of a human [24].

However, there are a few studies of AQP2 and AVPR2 in companion animals, including dogs and cats [1,8]. Moreover, the relationship between AQP2 and AVPR2 and the localization of these proteins’ expression in dogs with renal disease have rarely been investigated. As a result, we hypothesized that AQP2 and AVPR2 play an important role in water resorption in canine kidneys, just as they do in human and other animal species, by hypothesizing that the expression of AQP2 and AVPR2 was reduced in renal tissue in dogs with CKD.

The study aimed to know the expression of AQPs and AVPR2 in the kidneys of a canine with CKD.

## Materials and Methods

### Ethical approval

The Animal Ethics Committee of Faculty of Veterinary Medicine, Chiang Mai University approved the use of carcasses and all methods (License number R7/2560).

### Study period and location

The study was conducted from February 2019 to January 2021. The study was conducted at the Faculty of Veterinary Medicine, Chiang Mai University, Chiang Mai, Thailand.

### Sample collection

Two donated carcasses of dogs were used in this study. The first dog was a 4-year-old mixed breed with normal kidney tissue, whereas the second one was a 10-year-old Pomeranian dog with chronic renal disease. All carcasses were received from Small Animal Teaching Hospital, Faculty of Veterinary Medicine, Chiang Mai University. Two cadavers’ left and right kidneys were taken within 24 h of death and kept in 10% buffered formalin for 24 h before being analyzed.

### Sample preparation

Four kidneys were analyzed (left and right kidney in normal dog and left and right kidney in the dog with CKD). The kidney tissue was selected in the same area and then cut into the block. All kidney tissues were then fixed in buffered formalin and embedded in paraffin before being sectioned at 2 μm on a rotary microtome. To explore morphological changes and histochemical studies, the tissue section was stained with hematoxylin and eosin (H & E) and prepared for immunohistochemistry.

### Immunohistochemistry

The kidney tissue sections were deparaffinized with xylene and rehydrated with 95% ethanol. The sections were boiled in the antigen retrieval solution (1 mM Tris, pH 6.0) for 3 min. Endogenous peroxidase was blocked with 35% H_2_O_2_ in anhydrous methanol for 30 min, and non-specific binding was blocked with normal horse serum in phosphate-buffered saline (PBS; pH 7.4) for 30 min and then washed with PBS 3 times for 10 min. These sections were then incubated overnight at 4°C with the primary antibodies, anti-AQP-2 (ab15116, Abcam, UK) diluted 1:500, and anti-vasopressin receptor-2 (Anti-AVPR2, ab188748, Abcam) diluted 1:200 in PBS with 0.05% NaN_3_. The sections were then rinsed in PBS buffer for 10 min before being incubated for 1 h at 25°C with horseradish peroxidase-conjugated anti-rabbit secondary antibody (ab205718, Abcam) diluted 1:1000 in PBS. After increasing with PBS wash buffer 3 times, 10 min each time, the antibody-antigen response sites were visualized using browning chromogen by 1 min incubation with 0.05% 3, 3’-diaminobenzidine tetrachloride (Dako, Denmark), which is dissolved in distilled water with 0.1% H_2_O_2_, then counterstained with Mayer’s hematoxylin. After dehydration, the covers were mounted with a permanent mounting medium. Sections were viewed using a compound optical microscope (Olympus BX53; Olympus Corporation, Tokyo, Japan). Semi-quantitative immunohistochemistry involved using free software ImageJ Fiji (version 1.2; WS Rasband, National Institutes of Health, Bethesda, MD, USA) to study protein expression and localization in kidney tissue, masking the area of protein expression (brown color) and counting masks for percentage of area to study protein expression and localization [25,26].

### Statistical analysis

Data were expressed as mean±standard deviation(SD). Protein expression and localization were compared between the two groups using one-way analysis of variance with *post hoc* Mann–Whitney U-test was performed using GraphPad Prism 7.0 (GraphPad Software, USA). Statistical significance was defined as p<0.001.

## Results

**Figure-1:**
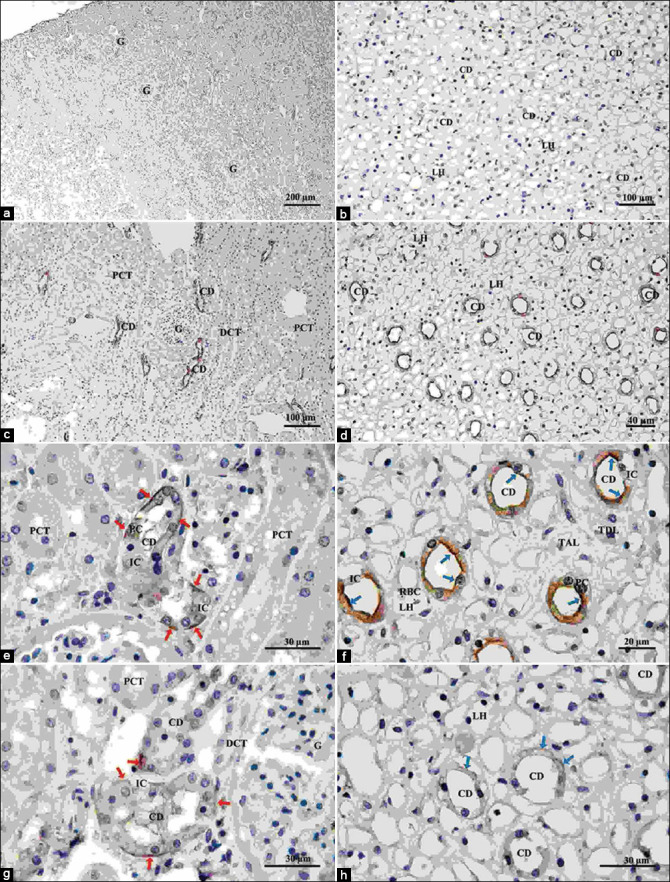
Low and high magnification of histological and immunohistochemical localization of aquaporin (AQP) 2 and arginine vasopressin receptor (AVPR) 2 in the cortex and medulla. Negative control in the cortex (a) and medulla (b). Immunolocalization immunoperoxidase labeling of AQP2 in the cortex (c and e, red arrow) and medulla (d and f, blue arrow). Immunolocalization immunoperoxidase labeling of AVPR2 in the cortex (g, red arrow) and medulla (h, blue arrow). The proteins’ localization and expression were shown in brown. AQP2 was found in the basolateral and apical membranes of the principal cells, whereas AVPR2 was found in the basolateral membrane of the principal cells in the renal cortex and the renal medulla. CD=Collecting duct, DCT=Distal convoluted tubule, G=Glomerulus, IC=Intercalated cell, LH=Thin segment Henle’s loop, PC=Principal cell, PCT=Proximal convoluted tubule, RBC=Red blood cell, TAL=Thick ascending Henle’s loop, TDL=Thick descending Henle’s loop.

Figure-1 (a-h) shows low and high magnification of renal histology and immunohistochemical localization of AQP2 and AVPR2 in the normal dog kidney in each kidney section; cortex and medulla compared with negative controls in the renal cortex (Figure-1a) and medulla (Figure-1b). Immunohistochemical staining of AQP2 in the renal cortex revealed a strong expression of AQP2 (red arrowhead) in the basement membrane of the principal cell of the collecting duct (Figures-1c and e). In contrast, immunoperoxidase labeled AQP2 immunohistochemistry in the medulla; overexpression of AQP2 (red arrowhead) at the intracellular and apical membrane of principal collecting duct cells (Figures-1d and f). Furthermore, immunolocalization and immunoexpression of AVPR2 (blue arrowhead) was found mostly in the collecting duct principal cells’ basolateral membrane, both in the cortex (Figure-1g) and medulla (Figure-1h).

**Figure-2:**
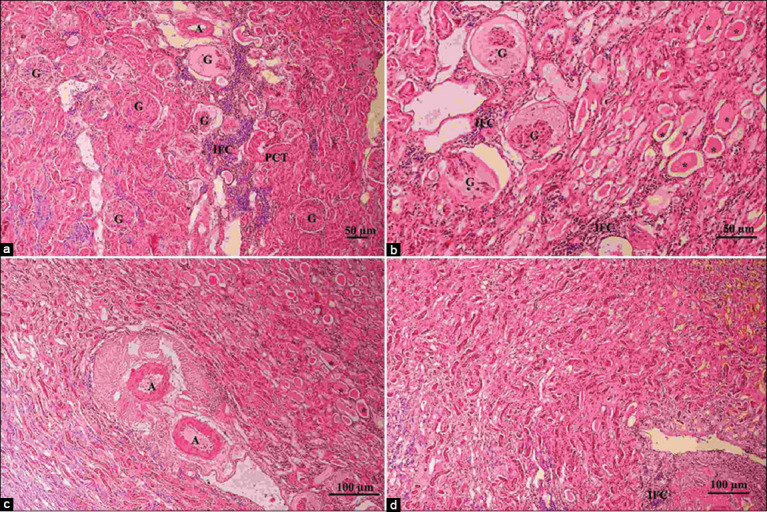
Light micrographs of hematoxylin and eosin (H and E) stained the kidney sections from canine kidney with chronic kidney disease (CKD) revealed the histological structure in the renal cortex (a-c) and medulla (d). Tubules with severe degenerative changes and damaged glomeruli. The glomeruli showed sclerosis, with many inflammatory cells infiltrating (a-b and d), the arteries were thickened (c), renal tubules were dilated and filled with pink casts to show renal thyroidization (star-shaped) (b and c), and tubular atrophy and interstitial fibrosis (d) (H and E, ×200). A=Artery, G=Glomerulus, IFN=Inflammatory cells, PCT=Proximal convoluted tubule, *=Pink casts.

Light micrographs of H & E stained kidney sections from canine kidneys with CKD are shown in Figures-2a-d. The histological structure of the renal cortex (Figures-2a-c) and medulla (Figure-2d) in CKD was revealed. Figure-2 shows the severe progressive degeneration and damage of the renal tubules and the glomeruli.

The glomeruli were sclerosis (glomerulosclerosis), there were many inflammatory cells infiltrated among the interstitial tissues (Figures-2a,b and d), the arterial walls appeared thickened (Figure-2c), and the renal tubules were dilated and filled with pink casts, indicating renal thyroidization (colloid-like hyaline cast formation) (star-shaped) (Figures-2b and c) and the renal tubules showed degeneration and atrophy, as well as interstitial fibrosis (Figure-2d).

**Figure-3:**
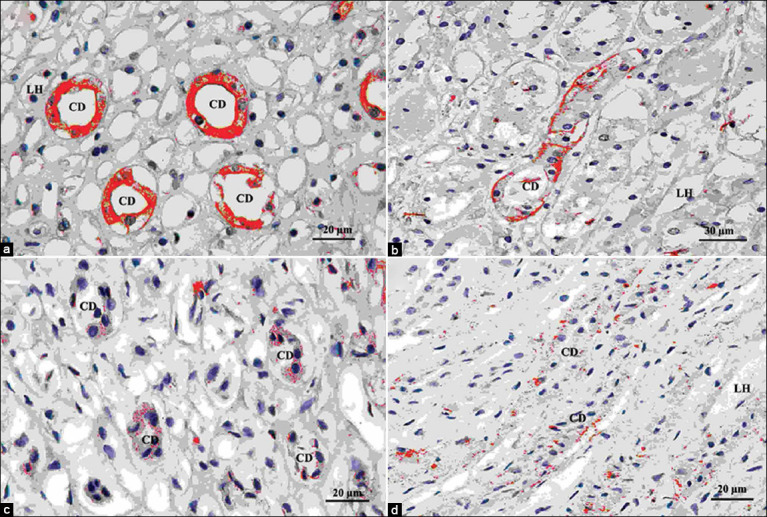
High magnification images were shown of immunolabeling aquaporin (AQP) 2 and arginine vasopressin receptor (AVPR) 2 in normal canine kidney (a and b) and were compared to immunolabeling AQP2 and AVPR2 in chronic kidney disease (CKD) (c and d). The protein localization and expression were shown in red color. The immunolabeling revealed that AQP2 (c) and AVPR2 (d) expressions were lower in canines with CKD when compared to AQP2 (a) and AVPR2 (b) expression in normal kidney. CD=Collecting duct, LH=Thin segment Henle’s loop.

Furthermore, immunolabeling of AQP2 and AVPR2 in the inner medulla of normal kidneys is shown in Figure-3. AQP2 expression was prominent in the apical plasma membrane and intracellular of the collecting duct principal cells (Figure-3a). The expression of AVPR2 was also high in the collecting duct principal’ basolateral membrane (Figure-3b). In contrast, immunolabeling with AQP2 (Figure-3c) and AVPR2 (Figure-3d) was less staining in canines with CKD (in red color).

Besides that, we used semi-quantitative immunohistochemistry to analyze protein expression and location within renal tissues. Data are presented as the mean±standard error of the mean (SEM) to represent protein expression and localization between CKD and normal kidney. Table-1 revealed that the number of protein expression of AQP2 in normal kidney was 5.062±0.4587, while the percentage of protein expression of AQP2 in CKD kidney was 1.218±0.1719, and the percentage of protein expression of AVPR2 in normal kidney was 4.306±0.7695, while the percentage of protein expression of AVPR2 in CKD kidney was 0.8536±0.1396.

**Table 1:** The semi-quantitative evaluation of protein AQP2 and AVPR2 expression (percent area) as the mean±SEM on kidney with CKD and normal kidney (NORM). The Mann–Whitney U-test: Values are given as mean±SEM (p<0.001).

Protein expression (%area)	AQP2	AVPR2
Kidney with CKD	1.218±0.1719	0.8536±0.1396
NORM	5.062±0.4587	4.306±0.7695

AQP2=Aquaporin 2; AVPR2=Arginine vasopressin receptor 2; SEM=Standard error of the mean; CKD=Chronic kidney disease

**Figure-4:**
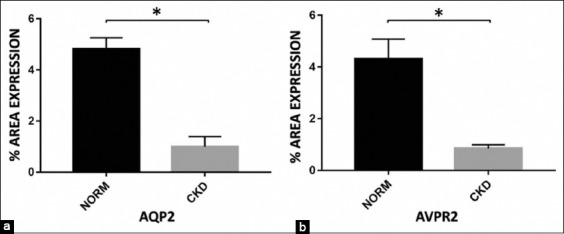
(a and b) Semi-quantitative analysis of aquaporin (AQP) 2 and arginine vasopressin receptor (AVPR) 2 immunostaining in canines with chronic kidney disease (CKD) and normal kidney (NORM). The Mann–Whitney U-test: Values are expressed as mean±SEM, with *p<0.001 significance level. Semi-quantitative analysis of CKD revealed a significant decrease in the percentage of protein expression in AQP2 and AVPR2.

Moreover, the percentage of protein expression revealed a CKD-related decrease AQP2 expression levels corresponding to a 4-fold decrease (Figure-4a, p<0.001). In parallel, in CKD, there was a 5-fold decrease in AVPR2 labeling (Figure-4b, p<0.001). When compared to the normal kidney, this result revealed a significant decrease in the percentage of protein expression in AQP2 and AVPR2 labeling in CKD.

## Discussion

In our study, we discovered the presence of AQP2 and AVPR2 in canine kidney’s principal cells. In this study, AQP2 was found to be strongly stained in the basolateral and apical membranes of the principal cells of the collecting duct, as well as in the renal cortex and medulla. According to a previous study of immunohistochemical on immunogold electron microscopy in normal and arginine vasopressin-deficient Brattleboro rats, AQP2 was localized in the basolateral membrane along the connecting tubules and collecting ducts [27]. Furthermore, previous research discovered that AVPR2 was strongly stained primarily in the basolateral membrane of the principal cells in the collecting duct in both the renal cortex and the renal medulla [11,28].

Furthermore, previous research concluded that increasing plasma osmolality could provoke the posterior pituitary gland to start releasing arginine vasopressin, leading to greater AQP2 expression in the apical plasma membrane of collecting duct cells [13]. When arginine vasopressin binds to AVPR2, the active form of PKA phosphorylates serine sites on the cytoplasmic C-terminus of the three AQP2 monomers, causing AQP2 protein to be released from vesicles and increasing AQP2 protein synthesis in the apical plasma membrane [29]. In response to arginine vasopressin, the overall process increases the concentration of AQPs as well as the proportion of phosphorylated serine at the 256, 264, and 269 sites [30-32]. The level of phosphorylated Ser261 starts to rise in the absence of arginine vasopressin stimulation and starts to fall in the presence of arginine vasopressin treatment [33,34]. In addition, to that, AQP2 phosphorylation at the Ser269 site (AQP2pS269) was realized to be present only in the apical membrane [31], whereas AQP2pS256 was discovered in both the apical membrane and intracellular vesicles [30]. Furthermore, phosphorylation of AQP2 at Ser261 (AQP2pS261) has been discovered in the Golgi apparatus and lysosomes [35], in spite of the fact that phosphorylation of AQP2 at S264 has been found in both apical and basal cell membranes [32].

The antibodies for immunocytochemical analysis that recognized the total amount of AQP2 in the kidney were used in this study. As a result, the locations of AQP2 phospho-forms in the collecting ducts of canine kidneys could be determined. Based on the literature, we believe that the strong expression shown in the basolateral and apical membranes of principal cells in the collecting ducts of canine kidneys which were caused by AQP2pS256, AQP2p264 in the cortex, and AQP2p256, AQP2pS264, and AQP2pS269 in the medulla.

Accordingly, studies of AQP2 expression in rat and mouse kidneys revealed that a small amount of this protein was discovered in the basolateral membrane of the inner medullary collecting duct, particularly in principal cells [31,32,35], which is consistent with our findings. However, our study revealed more details that AQP2 staining on the basolateral membrane in the renal cortex part and staining on the apical membrane in the renal medulla part of the principal cell were markedly stained in our investigation.

Due to the obvious important roles of AQP2 in regulating water balance in mammals, more detailed research is required regarding understanding the role of AQP2 in water balance regulation in canines.

Furthermore, water imbalance regulated by AQP2-AVPR2 has been associated with a variety of pathophysiological states, most of which are associated with altered urinary concentrating ability, such as renal ischemia/reperfusion injury [36], cisplatin-induced nephropathy [37], gentamicin-induced nephropathy [38], urinary tract obstruction [39], nephrotic syndrome [40], chronic heart failure [41], and renal disease [42]. CKD was a chronic disease characterized by hypertension, urinary concentrating abnormalities, and electrolyte imbalances which including polyuria, hypercalcemia, hypernatremia, and hypokalemia caused by changes in AQP2 and AVPR2 expression [43-46]. These were complex conditions with a wide range of glomerular and tubular anomalies that contributed significantly to overall renal dysfunction, reinforcing the idea that collecting duct water reabsorption was impaired due to AQP2 suppression in post-ischemic kidneys [36] and reduced expression of AQP2 expression in polyuria hypercalcemia in rats [43]. Throughout the case of acute renal disease, the apical membrane and subapical vesicle fraction in the collecting duct had significantly reduced AQP2 expression, which was confirmed by an immunohistochemical study; this dysregulation could be responsible for the decrease in AQP2 protein expression and impairment of AQP2 trafficking in the collecting duct [47].

As a result, CKD caused a significant change in the AVPR2 protein in the nephrectomy group compared to the control group in rat kidney with experimental nephrectomy models, and the findings revealed that CKD caused by nephrectomy was strongly associated with polyuria and a vasopressin-resistant decreased expression of AQP2 [48], which was similar to our findings. The rat with hypercalcemia and hypokalemia had vasopressin-resistant polyuria in urinary concentrating ability [45,49] and increased prostaglandin production [50], there were decreased adenylate cyclase and cAMP (the second messenger for vasopressin action), resulting in decrease in AQP2 expression [13]. A net loss of water due to the insufficient insertion of the AQP2 water channel into the apical membrane of collecting duct cells appears to be a typical condition of hypernatremia [51].

In addition, previous research in hypertensive Sprague-Dawley rats has shown that AQP2 expression and cAMP concentration in the renal medulla are decreased [52,53]. Rising blood pressure counteracts encourage water excretion, resulting in decreased arginine vasopressin sensitivity [52,53] and/or excess nuclear factor κB (NF-κB) activity in the renal medulla collecting duct level [52]. The major lipopolysaccharide-activated pro-inflammatory NF-κB affects the expression of AQP2 in the collecting tube, demonstrating that AQP2 mRNA and protein levels are decreased; this study and subsequent research demonstrated that NF-κB was a key biological modulator of AQP2 transcription [54]. In regards, immunohistochemistry and RT-PCR experiments in renal disease confirmed a nearly significant decrease in AVPR2 protein and mRNA expression [55]. Immunocytochemistry and immunoelectron microscopy revealed that the level of AQP2 in the principal cells was reduced, implying that the lower level of AQP2 could be a significant factor contributing to the changes in the water permeability of the collecting duct and the decrement in vasopressin response in patients with CKD [12], which appears to be strongly found in a manner similar to our immunohistochemistry.

There has recently been evidence supporting the efficacy and safety of the AQP2 antagonist (tolvaptan) in the treatment of hyponatremia caused by renal function in patients with acute or chronic heart failure [56]. In other words, AQP2 may be a useful marker for estimating tolvaptan responses, notably in CKD patients. In addition, a study was conducted in which urine AQP2 (U-AQP2) was used as an innovative non-invasive marker to predict responses to tolvaptan in humans [57,58]. Urine AQP2 excretion in dogs can still be used as a prognostic tool to differentiate polyuria diseases such as central or NDI, inappropriate arginine vasopressin protein release, and primary polydipsia [34].

## Conclusion

The study shows the precise localization of AQP2 and AVRP2 in canine kidneys, as well as the low expression of AQP2 and AVRP2 in nephrotic syndrome. This could refer to the loss of water transport and the body’s ability to maintain water homeostasis. Changes in the location and expression of AQP2 and AVRP2 also contribute to diseases, such as CKD, polyuria, and NDI. Scientific studies appeared to be destined to clarify their roles, as well as the relationship between AQP2 and AVRP2 underlying water balance abnormalities (such as liver cirrhosis and congestive heart failure). Furthermore, it may pave the way for more targeted therapeutic interventions with AQP antagonist drugs in the veterinary field.

## Authors’ Contributions

PM, WP, and RM: Study concept and conducted the study. PM and PK: Collected the sample. PM and WP: Formal analysis and data curation. PM and RM: Drafted the manuscript. PM, WP, and RM: Provided scientific advice, review, and editing. All authors read and approved the final manuscript.
